# Acer Truncatum Seed Oil Alleviates Learning and Memory Impairments of Aging Mice

**DOI:** 10.3389/fcell.2021.680386

**Published:** 2021-05-14

**Authors:** Xiao Li, Ting Li, Xiao Yue Hong, Jian Jun Liu, Xi Fei Yang, Gong Ping Liu

**Affiliations:** ^1^Key Laboratory of Ministry of Education of China and Hubei Province for Neurological Disorders, Department of Pathophysiology, School of Basic Medicine and the Collaborative Innovation Center for Brain Science, Tongji Medical College, Huazhong University of Science and Technology, Wuhan, China; ^2^Department of Laboratory Medicine, Zhongnan Hospital of Wuhan University, Wuhan University, Wuhan, China; ^3^Key Laboratory of Modern Toxicology of Shenzhen, Shenzhen Medical Key Discipline of Health Toxicology (2020-2024), Shenzhen Center for Disease Control and Prevention, Shenzhen, China; ^4^Co-innovation Center of Neuroregeneration, Nantong University, Nantong, China

**Keywords:** aging, acer truncatum seed oil, proteomics, learning memory, BDNF

## Abstract

Aging, characterized by a time-dependent functional decline of physiological integrity, is the major independent risk factor for many neurodegeneration diseases. Therefore, it’s necessary to look for natural food supplements to extend the healthy lifespan of aging people. We here treated normal aging mice with acer truncatum seed oil, and found that the seed oil significantly improved the learning and memory ability. Proteomics revealed that the seed oil administration changed many proteins expression involving in biological processes, including complement and coagulation cascades, inflammatory response pathway and innate immune response. BDNF/TrkB signaling pathway was also activated by acer truncatum seed oil treatment. And the seed oil administration increased the expression of postsynaptic related proteins including PSD95, GluA1, and NMDAR1, and decreased the mRNA level of inflammatory factors containing IL-1β, TNF-α, and IL-6. These findings suggest that acer truncatum seed oil holds a promise as a therapeutic food supplement for delaying aging with multiple mechanisms.

## Introduction

Aging, characterized by a time-dependent functional decline of physiological integrity (Lopez-Otin et al., 2013), is a major independent risk factor for many neurodegeneration diseases such as Alzheimer’s disease (AD), Parkinson’s disease, Amyotrophic lateral sclerosis, and so on. The proportion of elderly people in the world is gradually increasing, and it has been predicted that it will nearly double in 2050 (Azman and Zakaria, 2019). However, the research related to aging is still a small field relative to the age-related diseases (Partridge et al., 2020). Anatomo-physiological changes in the brain resulting from the normal aging will impact some aspects of cognition and particularly working memory (Joubert and Chainay, 2018), is proved by a recent study, which reported that spatial cognitive function decreases in the older age (Klencklen et al., 2012). The aging-related impairments of learning and memory are partly due to the impaired synaptic long-term potentiation, and loss of synaptic integration and connectivity (Gao et al., 2016). Moreover, DNA damages including somatic mutations, copy number variations and chromosomal aneuploidies have been found to be connected with aging (Faggioli et al., 2012; Forsberg et al., 2012; Moskalev et al., 2013). Accumulated data strongly suggest that the upregulation of chronic inflammation induced by an age-related redox imbalance, will activate the expression of many pro-inflammatory factors such as interleukin-1β (IL-1β), interleukin-6 (IL-6), and tumor necrosis factor (TNF-α) (Cho et al., 2003; Chung et al., 2009). Furthermore, chronic inflammation also plays important roles in the pathogenesis of many age-related diseases, such as cancer, obesity, AD and metabolic syndrome (Chung et al., 2006). Interventions to delay aging may extend the healthy lifespan of people (Strong et al., 2016).

There are many natural products which are found to delay aging and benefit cognition. For example, Omega-3 polyunsaturated acids including α-linolenic acid, eicosapentaenoic acid and docosahexaenoic acid (DHA), increased synaptic plasticity and had anti-inflammatory effects even at old age (Cutuli, 2017). Nervonic acid had significant biological functions, such as promoting brain development, alleviating memory impairment, and delaying brain aging (Li et al., 2019). Moreover, long-term dietary α-linolenic acid supplement improved learning and memory impairments of naturally aging rats (Gao et al., 2016). The moderate aerobic exercise with vitamin E (VE) supplement increased antioxidant enzyme activities in the hippocampus of aged rats (Devi and Kiran, 2004), with reduced oxidative damage of proteins (Jolitha et al., 2006), and improved learning ability (Jolitha et al., 2009). The inflammatory responses induced by palmitic acid treatment in the microglial cells were effectively reversed by linoleic acid (Tu et al., 2019). We also found that supplementation of folate/vitamin B12 or betaine significantly reversed the memory deficits of a chronic hyperhomocysteinemia model or aged rats, respectively (Wei et al., 2011; Chai et al., 2013).

As a food supplement, the acer truncatum seed oil may play a positive role in anti-aging. Herein, we treated 20-month-old aging mice with acer truncatum seed oil for 1 month, and tested the effects of acer truncatum seed oil on the learning and memory abilities. We found that the oil significantly alleviated the cognitive impairments of aging mice. Proteomic analysis revealed that, 44 increased differentially expressed (DE) proteins in the aging control mice, which associated with biological process including complement and coagulation cascades, inflammatory response pathway and innate immune response, was markedly reversed by oil administration. Moreover, the brain-derived neurotrophic factor (BDNF) signal pathway was activated and the decline of postsynaptic protein expression was also reversed with attenuation of gene expression of IL-1β, IL-6, and TNF-α in the hippocampus. The data suggested that acer truncatum seed oil may be a potential diet supplements for preventing aging.

## Materials and Methods

### Animals and Treatment

Two-month old male C57/BL6 mice were purchased from the Guangdong Medical Laboratory Animal Center (Guangdong, China). Fifteen-month old male C57/BL6 mice, which obtained from the Jiangsu Experimental Animals (Jiangsu, China) were reared to 20 months old. All the animals were provided with adequate food and water, and kept in a standard environment with temperature (22 ± 2°C) and a 12 h light-dark cycle. All experimental procedures were carried out in accordance with the National Institutes of Health guide for the care and use of Laboratory animals (NIH Publications No. 8023, revised 1978) and were approved by the Ethics Committee of the Shenzhen Center for Disease Control and Prevention.

All mice aged 2 months or 20 months were randomly divided into control or acer truncatum seed oil treatment group (9 mice in each group). The control or oil-treated mice were orally treated with saline (0.01 ml/g/day) or acer truncatum seed oil (0.01 ml/g/day) for 1 month, respectively. Acer truncatum seed oil (jinfenglu) was provided by Yunnan Jinfeng Biotechnology Co., Ltd. After treatment for 1 month, the mice were subjected to detect cognitions by Morris water maze test.

### Morris Water Maze (MWM)

The spatial learning and memory of mice was detected by the MWM (Morris et al., 1982). The temperature of the MWM room was kept at 24 ± 2°C. Before each experiment (2 h before), all mice were placed in the experiment room to allow them to adapt to the environment. All animals were trained in a circular pool (58 cm high and 122 cm in diameter) to find a hidden platform for 5 consecutive days, 4 trials per day with a 30 s interval from 13:00 to 18:00 p.m. The circular pool filled with 23 ∼ 25°C opaque water, was divided into four quadrants, and the escape platform (12 cm in diameter) was put in the third quadrant. The water surface was about 1.2 cm higher than the escape platform. On each trial, the mice started from one of the four quadrants facing the wall of the pool and ended when the animal climbed on the platform. If the mice found the platform within 60 s, we recorded the time and then kept the mice stay on the platform for another 30 s. If the animals failed to find the platform within 60 s, we recorded the time as 60 s. Then these mice were guided onto the platform and stayed on the platform for 30 s. The spatial memory was tested after 24 h of completing 5 days training. The escape platform was removed during the detection. The time that the mice first arrived on the removed platform was recorded. Moreover, other parameters including the number of times to cross the platform, swimming speed and swimming distance were automatically recorded by a video camera fixed to the ceiling, 1.5 m from the water surface. The camera was connected to a digital-tracking device attached to an IBM computer.

## Proteomic Analysis

### Preparation of Protein Samples

The hippocampal tissues were lysed in 8M Urea with ultrasonic homogenizer, and then, kept on ice for 30 min. The lysates were centrifuged at 12,000 g, 4°C for 30 min followed by collected the supernatant. The protein concentration was measured with a BCA protein assay kit (Thermo Fisher Scientific, NJ, United States).

### Tandem Mass Tag (TMT) Labeling

Samples were labeled by TMT according to the reported method (Xu et al., 2019). The sample with 100 μg protein was incubated with 10 mM dithiothreitol at 37°C for 1 h. Then, iodoacetamide was added into the sample, and its final concentration was 25 mM. Samples were diluted to 1M Urea with phosphate buffered saline (pH = 8.0), digested with 1:25 w/w trypsin at 37°C for 15 h. The digestion reaction was terminated with 1% formic acid followed by centrifugation. The supernatants were desalted in reversed-phase column (Oasis HLB; Waters, Milford, MA, United States), then dried in a vacuum concentrator, and finally dissolved in triethylammonium bicarbonate. Peptides were incubated with TMT label for 1 h: the young control group was labeled with TMT-126, the young oil treated group was labeled with TMT-127, the aging control group was labeled with TMT-128, and the aging oil treated group was labeled with TMT-129 (*n* = 4 per group). The reaction was terminated by hydroxylamine. Different TMT label samples were mixed into four tubes. The labeled peptides were desalted, dried, and finally dissolved in 100 μL 0.1% FA.

### High Performance Liquid Chromatography (HPLC) Separation

The labeled peptides were fractionated according to a previously described method (Wang et al., 2019). Specifically, four mixed peptides were separated in the Xbridge BEH300 C18 column (Waters Milford, MA, United States) by HPLC (UltiMate 3000 UHPLC; Thermo Fisher Scientific, Waltham, MA, United States). 15 fractions that obtained from every peptide, were dried, and dissolved in 20 μL 0.1% FA followed by further liquid chromatography (LC)-mass spectrometry (MS)/MS analysis.

### LC-MS/MS Analysis and Database Searching

Peptide analysis was conducted according to previous research methods (He et al., 2019). The analytical capillary column (Upchurch, Oak Harbor, WA, United States) packed with C18 silica resin (Varian, Lexington, MA, United States) was used to separate peptides. We used the quadrupole Orbitrap mass spectrometer (Q-Exactive, Thermo Scientific, United States) to collect and analyze ionized peptides. All raw mass spectra were searched with the Uniprot-Mus musculus database (released in November, 2019) by Proteome Discoverer 2.1 software (Thermo Fisher Scientific, Waltham, MA, United States). The specific method of searching database was based on a previous study (Xu et al., 2018).

### Bioinformatics Analysis

The analysis of proteomic results was carried out by multiple approaches. Gene ontology (GO) annotation enrichment analysis of differentially expressed (DE) proteins was analyzed at database for annotation, visualization and integrated discovery (DAVID) version 6.8^1^. We used RStudio software to complete the omics heat map drawing and cluster analysis. Molecular Complex Detection (MCODE) analysis was implemented in the Cytoscape version 3.7.1. The mass spectrometry proteomics data have been deposited to the ProteomeXchange Consortium (Ma et al., 2019).

### Western Blotting

The mice hippocampal tissues were homogenized, sonicated, and then centrifuged at 12,000 g for 30 min at 4°C. Protein concentration was detected using a BCA protein assay kit (Thermo Fisher Scientific, NJ, United States). All lysates samples were separated by 8∼12% PAGE Gel Fast Preparation Kit (Epizyme Biotechmology). Proteins were transferred to the methanol infiltrated polyvinylidene fluoride (PVDF) membrane after separation. Non-specific binding sites in the PVDF membrane were blocked by incubating with 5% skim milk followed by antibody incubation: STAT1 (1:1,000, proteintech), CD9 (1:1,000, abcam), FINC (1:1,000, abcam), BDNF (1:1,000, abcam), DM1A (1:1,000, Sigma), p-TrkB (1:1,000, Sigma), TrkB (1:1,000, Biovision), p-ERK (1:1,000, Cell Signaling Technology), ERK (1:1,000, proteintech), p-Akt (1:1,000, Cell Signaling Technology), Akt (1:1,000, Cell Signaling Technology), p-CREB (1:1,000, Cell Signaling Technology), CREB (1:1,000, Cell Signaling Technology), PSD95 (1:1,000, abcam), GluNR1 (1:1,000, abcam), GluA1(1:1,000, abcam), Synaptophysin (1:1,000, abcam), Synapsin-II (1:1,000, abcam). Secondary antibody goat anti-Mouse (1:10,000, Invitrogen) and goat anti-Rabbit (1:10,000, Invitrogen) were used. Western blotting images were obtained by ECL exposure and quantified by ImageJ software.

### Quantitative Real-Time PCR

Total RNA was extracted from hippocampus using Trizol (Invitrogen, 15596026, United States). Reverse transcription and real-time quantitative PCR were accomplished by Takara kit. 1 μg of total RNA was used to synthesize cDNA. The expression level of the objective gene was normalized by glyceraldehyde-3-phosphate dehydrogenase (GAPDH). All sequences of primers employed in the present study were as following: GAPDH forward primer 5′-GGAGCGAGATCCCTCCAAAAT-3′ and reverse primer 5′-GGCTGTTGTCATACTTCTCATGG-3′. IL-1β forward primer 5′- GCACTACAGGCTCCGAGATGAA-3′ and reverse primer 5′-GTCGTTGCTTGGTTCTCCTTGT-3′. TNF-α forward primer 5′-CACGCTCTTCTGTCTACTGAACTTC-3′ and reverse primer 5′-ATGATCTGAGTGTGAGGGTCTGG-3′. IL-6 forward primer 5′-AGTGGCTAAGGACCAAGAC-3′ and reverse primer 5′- ATAACGCACTAGGTTTGCCGA-3′. IL-1α forward primer 5′-CGAAGACTACAGTTCTGCCATT-3′ and reverse primer 5′-GACGTTTCAGAGGTTCTCAGAG-3′. IL-4 forward primer 5′- GGTCTCAACCCCCAGCTAGT-3′ and reverse primer 5′- GCCGATGATCTCTCTCAAGTGAT-3′. IL-13 forward primer 5′- CCTGGCTCTTGCTTGCCTT-3′ and reverse primer 5′- GGTCTTGTGTGATGTTGCTCA-3′.

### HE Staining

The tissue slices were attached to a glass slide and dried for 30 min. The slices were stained with hematoxylin for 5 min, rinsed with tap water to remove excess staining solution for 10 min, and then washed again with distilled water. They were differentiated by 0.5% hydrochloric acid ethanol for 10 s, and then rinsed with tap water for 10 min. The slices were further stained with eosin for 1 min, and Gradient ethanol dehydration: 70% ethanol for 10 s, 80% ethanol for 10 s, 90% ethanol for 10 s, and absolute ethanol for 10 s. Finally, these tissue slices were transparentized with xylene for 10 min, and then sealed with neutral gum.

### Statistical Analysis

All data were presented as mean ± standard errors (SEM). Statistical tests were used two-way repeated measures analysis of variance (ANOVA) tests or one-way ANOVA followed by Tukey’s multiple comparison tests with the GraphPad Prism 8 (GraphPad Software, United States). *P* < 0.05 was considered as statistically significant difference.

## Results

### Acer Truncatum Seed Oil Attenuated Learning and Memory Impairments of Aging Mice

**FIGURE 1:**
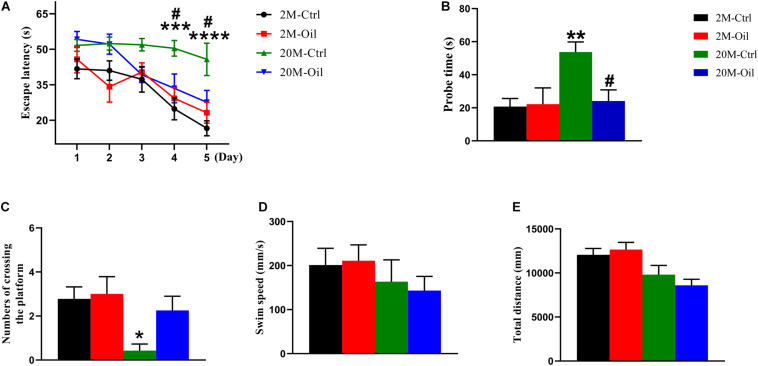
Acer truncatum seed oil ameliorated cognitive deficits of aging mice. **(A)** Oil treatment improved learning ability of aging mice shown by decreased escape latency during training phase detected by Morris water maze. Two-way repeated measures ANOVA tests followed by Tukey’s multiple comparison tests, ***p < 0.001, ****p < 0.0001 (2M vs. 20M); ^#^*p* < 0.05 (20M vs. 20M-Oil). **(B)** Oil treatment improved the memory capacity shown by the decreased escape latency of aging mice during the test. One-way ANOVA followed by Tukey’s multiple comparison tests, **p < 0.01 (2M vs. 20M); ^#^*p* < 0.05 (20M vs. 20M-Oil). **(C)** The number of times that aging mice crossed the platform was significantly decreased than that of young mice. One-way ANOVA followed by Tukey’s multiple comparison tests, *p < 0.05 (2M vs. 20M). There was no significant difference in swimming speed **(D)** or total swimming distance **(E)** among the 4 groups. One-way ANOVA followed by Tukey’s multiple comparison tests. All data were expressed as mean ± SEM, *n* = 7–9 mice for each group.

The main ingredients of acer truncatum seed oil were listed in Table 1, which included eicosapentaenoic acid, DHA, nervonic acid, linoleic acid, α-linolenic acid and vitamin E, which all reported to benefit for the cognition. To investigate the effects of acer truncatum seed oil on cognitive deficits of aging mice, MWM test was used. During the training phase, we found that the latency of the aging control mice probing for the hidden platform was significantly longer compared with the young control (at 4th and 5th d) (Figure 1A). However, acer truncatum seed oil treatment significantly induced the aging mice to spend less time to find the platform at 4th and 5th d (Figure 1A). During the testing phase, the aging control mice took longer time to reach the site where the platform placed in the training phase (Figure 1B), and showed a significant decrease in the number of crossing the platform than the young control (Figure 1C). However, the seed oil treatment remarkably reduced the escape latency of aging mice (Figure 1B). Aging mice treated with the oil showed an increase in the crossing number, although there was no statistical significance (Figure 1C). The swimming speed and swimming distance of all experimental mice had no significant difference (Figures 1D,E), which excluded motor deficits. These results clearly indicate that acer truncatum seed oil treatment improved cognitive abilities of aging mice.

**TABLE 1:** The ingredients and contents of the acer truncatum seed oil.

Category	Name	Content/%
Monounsaturated fatty acid	Palmitoleic acid	0.096
	Oleic acid	23.01
	11-Eicosaenoic acid	2.17
	Erucic acid	16.24
	Nervonic acid	6.27
Polyunsaturated fatty acid	Linoleic acid	33.16
	γ-Linolenic acid	0.88
	α-Linolenic acid	7.40
	Arachidonic acid	0.29
	13,16-Docosadienoic acid	0.027
	Eicosapentaenoic acid	0.33
	Docosahexaenoic acid	0.17
Saturated fatty acid	Tetradecanoic acid	0.026
	Palmitic acid	3.88
	Heptadecanoic acid	0.087
	Stearic acid	2.39
	Arachidic acid	0.24
	Behenic acid	0.75
	Wood tar acid	0.029
Others	β-Carotene	183.7 μg/100 g
	Vitamin E	42.8 mg/100 g
	Iron	4.03 mg/kg

### Aging Induced Proteomic Profiles Change in the Hippocampus

**FIGURE 2:**
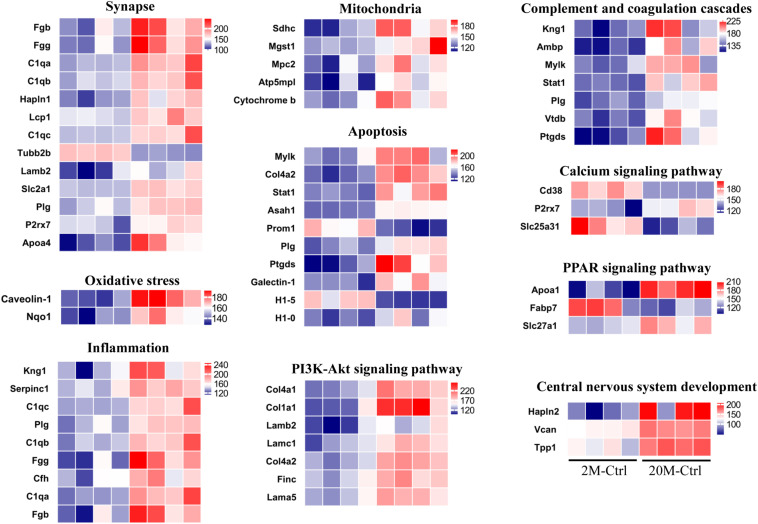
DE hippocampal proteins in aging vs. young control mice. The aging related proteins functionally classified according to uniport database. The color of blue represented low abundance, red represented high abundance (*n* = 4 mice/group).

In order to investigate the negative effects of aging in the proteomics, we analyzed protein expression in the hippocampus, and found that there were 121 DE proteins (Ratio < 1.2 or Ratio < 0.8, and *P* < 0.05) in the aging control mice compared with the young control (Supplementary Table 1). 121 DE proteins included 109 up-regulated proteins (red dots) and 12 down-regulated proteins (green dots) analyzed by the volcano plot (Supplementary Figure 1A). The DAVID version 6.8 was used for GO enrichment analysis, which shown that these proteins were involved in the related biological processes including extracellular matrix organization, complement activation, innate immune response and hemostasis, etc. (Supplementary Figure 1B). Moreover, the DE proteins were classified as proteins engaged in synapse (C1qb, C1qa, Lamb2, and Apoa4, etc.), oxidative stress (Caveolin-1 and Nqo1), inflammation (Kng1, STAT1, Plg, and Vtdb, etc.), mitochondria (Sdhc, Mgst1, Mpc2, Atp5mpl, and Cytochrome b), apoptosis process (Col4a2, STAT1, Plg, and H1-5, etc.), PI3K-Akt signaling pathway (Col4a1, Col1a1, Lamb2, and Col4a2, etc.), complement and coagulation cascades (C1qb, C1qc, and C1qa, etc.), calcium signaling pathway (Cd38, P2rx7, and Slc25a31), peroxisome proliferators-activated receptors (PPAR) signaling pathway (Apoa1, Fabp7, and Slc27a1) and central nervous system development (Hapln2, Vcan, and Tpp1) (Figure 2). MCODE analysis discovered closely linked versions of their protein-protein interaction (PPI) network, which included metabolism, negative regulation of endopeptidase activity, cell migration, complement, and coagulation cascades, complement activation, myelination, PI3K-Akt signaling pathway, autophagy, immune response, and mitophagy (Supplementary Figure 2).

### Acer Truncatum Seed Oil Treatment Changed the Proteomic Profiles in the Hippocampus of the Aging Mice

**FIGURE 3:**
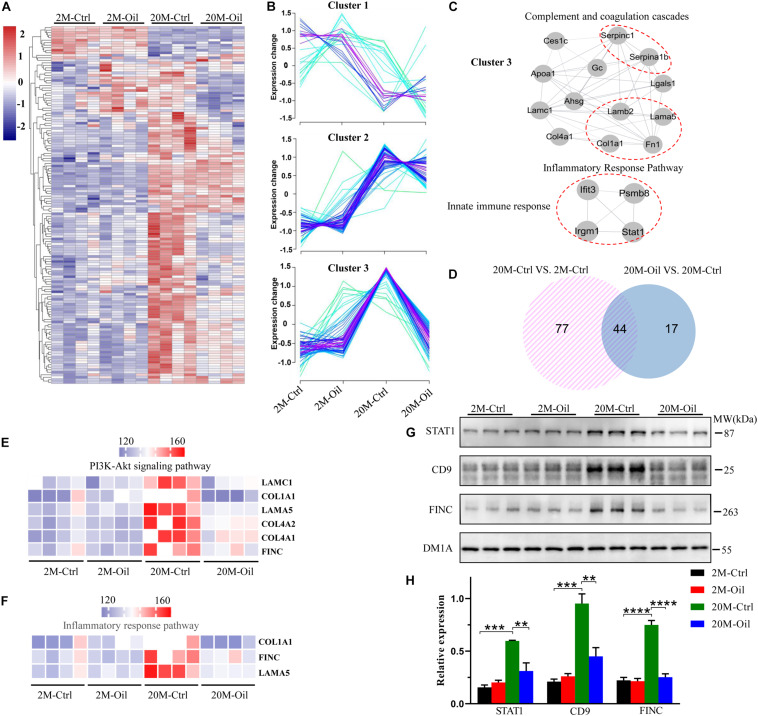
Proteomic profiles of the hippocampus in acer truncatum seed oil treated aging mice. **(A)** The heatmap analysis for all DE proteins. **(B)** All DE proteins were analyzed into three clusters by RStudio. **(C)** Detected PPI modules in cluster 3. **(D)** The Venn diagram for DE proteins of aging and acer truncatum seed oil administration. DE proteins of acer truncatum seed oil treatment mice in PI3K-Akt signaling pathway **(E)** and inflammatory response pathway **(F)**. Validation of selected DE proteins by western blotting with the loading control (DM1A) **(G,H)** One-way ANOVA followed by Tukey’s multiple comparison tests, **p < 0.01, ***p < 0.001, ****p < 0.0001. All data were expressed as mean ± SEM, *n* = 3 mice/group.

In order to explore the molecular mechanism of the seed oil improving learning and memory of aging mice, proteomics technology was used to analyze the proteins expression in the hippocampus. A total of 4,309 proteins were identified in the entire hippocampus, and heatmap analysis of all the DE proteins was shown in Figure 3A, which completed by RStudio software. Although acer truncatum seed oil administration induced only 11 DE proteins in the hippocampus of the young mice, we found that the oil treatment induced 61 DE proteins in the hippocampus of aging mice (Supplementary Table 2).

Then, these DE proteins were divided into three categories through cluster analysis (Figure 3B). Cluster 1 (*n* = 23) including proteins was mainly down-regulated in the aging control compared to the young control. The proteins in the cluster 2 (*n* = 54) were up-regulated in the aging control mice compared to the young control, while the oil treatment had no influence on their expressions. Relative to the young control, the proteins in the cluster 3 (*n* = 65) were up-regulated in the aging control, but they were significantly restored by the seed oil treatment. We also performed the MCODE analysis for the proteins in the cluster 3, which involved in three modules: complement and coagulation cascades, inflammatory response pathway and innate immune response (Figure 3C).

To further investigate the reversal effect of acer truncatum seed oil on protein expression in the aging mice, we drew a Venn diagram and found that the seed oil reversed the expression of 44 proteins in the aging control, which also has differential expression compared to the young control (Figure 3D). The volcano plot revealed that oil-treatment induced only 2 proteins up-regulation (red dots) and 59 proteins down-regulation (green dots) in the aging mice (Supplementary Figure 3A). The 10 most varied DE proteins were annotated in the Supplementary Figure 3A. These reversed proteins were mainly related to the phosphatidylinositide 3-kinases (PI3K) -Akt signaling pathway, which included laminin subunit gamma 1 (LAMC1), collagen alpha-1 (I) chain (COL1A1), laminin subunit alpha-5 (LAMA5), collagen alpha-1 (IV) chain (COL4A1), collagen alpha-2 (IV) chain (COL4A2), and fibronectin (FINC) (Figure 3E), and inflammatory response pathway, which contained COL1A1, FINC, and LAMA5 (Figure 3F). Furthermore, GO (gene ontology) enrichment analysis was used to obtain further insights into the biological process of 44 reversed proteins by seed oil treatment in the aging mice. The reversed proteins were in enriched function in extracellular matrix organization, angiogenesis, innate immune response, collagen-activated tyrosine kinase receptor signaling pathway and vitamin transport, etc. (Supplementary Figure 3B). STAT1, CD9, and FINC were up-regulated in the aging control mice, but they were reversed by seed oil treatment, which found by proteomics analysis and proved by western blotting (Figures 3G,H). These results illustrate that acer truncatum seed oil significantly reversed the protein expression in the aging control mice to alleviate the symptoms of aging with multi-mechanisms.

### Acer Truncatum Seed Oil Administration Activated BDNF Signaling Pathway

**FIGURE 4:**
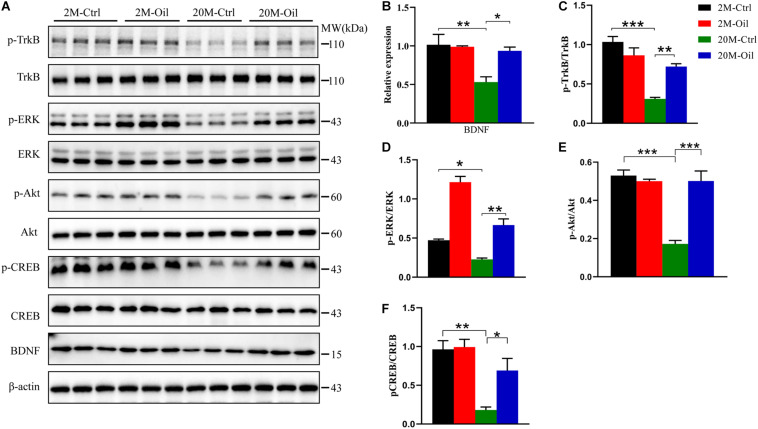
Acer truncatum seed oil administration activated BDNF signal pathway in aging mice. Compared with 2-month-old (2M-Ctrl) mice, the protein levels of BDNF, p-TrkB, p-ERK, p-Akt, and p-CREB decreased in 20-month-old (20M-Ctrl) mice, were reversed by oil treatment, which measured by western blotting **(A)**, and quantitative analyses **(B–F)**. One-way ANOVA followed by Tukey’s multiple comparison tests, *p < 0.05, **p < 0.01, ***p < 0.001. All data were expressed as mean ± SEM, *n* = 3 mice for each group.

The decreased BDNF signaling was related to memory impairment in aging (Erickson et al., 2012). Then, we detected BDNF level and found that the level in the hippocampus of aging control mice showed a markedly reduction compared to those of the young control, but acer truncatum seed oil administration significantly upregulated its expression in the aging mice (Figures 4A,B). BDNF binding tropomyosin receptor kinase B (TrkB) activated auto-phosphorylation on the tyrosine residues of TrkB in the intracellular region, resulting in the activation of multiple signaling cascades including the PI3K-AKT pathway, the phospholipase Cγ1 (PLCγ1)-PKC pathway and the MAPK (mitogen-activated protein kinases) -ERK (extracellular signal-regulated kinase) pathway (Longo and Massa, 2013; Guo et al., 2019). Therefore, we analyzed the phosphorylation level of TrkB, ERK, and AKT (p-TrkB, p-ERK, and p-AKT), and found that p-TrkB, p-ERK, and p-AKT levels remarkably decreased in the hippocampus of aging control, but the oil administration reversed the decline of these phosphorylated proteins (Figures 4A,C–E). Phosphorylated cyclic AMP response element-binding (p-CREB) was reported to be the transcriptional regulator of BDNF mRNA (Wu Y. et al., 2020). Furthermore, the decline of p-CREB protein expression in aging control mice was obviously reversed by the treatment with the oil (Figures 4A,F). These results suggest that BDNF signaling pathway may play an important role in the mechanisms of the oil administration improving learning and memory in aging mice.

### Acer Truncatum Seed Oil Increased Synapse-Related Proteins Expression in the Hippocampus of Aging Mice

**FIGURE 5:**
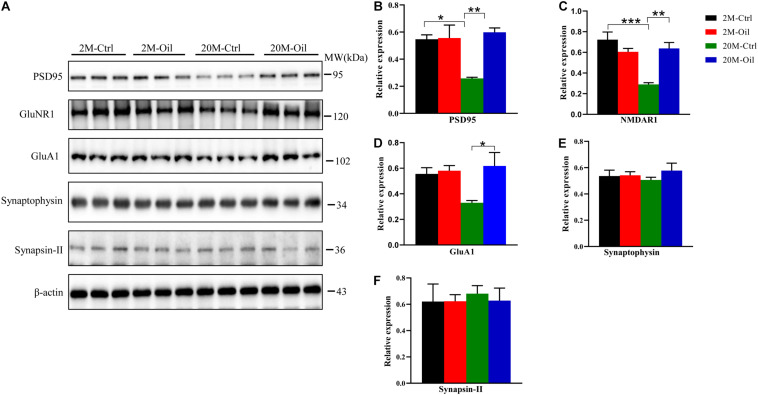
Acer truncatum seed oil increased expression of synapse-related proteins in aging mice. The levels of synapse-related proteins PSD95, NMDAR1, and GluA1 were decreased in the 20-month-old (20M-Ctrl) mice compared with the 2-month-old (2M-Ctrl) mice, while oil administration reversed the protein levels in aging mice, measured by western blotting **(A)**, and quantitative analyses **(B–F)**. One-way ANOVA followed by Tukey’s multiple comparison tests, *p < 0.05, **p < 0.01, ***p < 0.001. All data were expressed as mean ± SEM. *n* = 3 mice for each group.

Learning and memory are associated with the growth of preexisting and remodeling synapses (Bailey et al., 2015). Thus, synapse-related proteins were detected by western blotting. Postsynaptic markers including postsynaptic density protein 95 (PSD95), N-methyl D-aspartate receptor subtype R1 (GluNR1), AMPA-selective glutamate receptor 1 (GluA1) were significantly down-regulated in the aging control mice compared with the young control (Figures 5A–D). However, the seed oil markedly increased these postsynaptic proteins in aging mice (Figures 5A–D). Compared with the young control, no significant changes of presynaptic markers including synaptophysin and synapsin-II were observed in aging mice with the oil treatment or not (Figures 5A,E,F). These results demonstrate that acer truncatum seed oil increased the expression of several synapse-related proteins in the aging mice.

### Acer Truncatum Seed Oil Reduced Gene Expression of Inflammatory Cytokines

**FIGURE 6:**
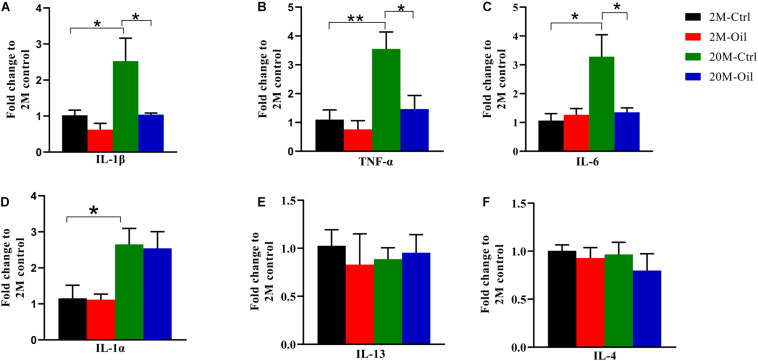
Acer truncatum seed oil reduced gene expression of inflammatory cytokines in aging mice. **(A–F)** The mRNA levels of inflammatory cytokines IL-1β, TNF-α, IL-6, and IL-1α were increased in hippocampus of 20-month-old (20M-Ctrl) mice compared with the 2-month-old (2M-Ctrl) mice detected by RT-qPCR, while oil treatment (20M-Oil) reversed expression. One-way ANOVA followed by Tukey’s multiple comparison tests, *p < 0.05, **p < 0.01. All data were expressed as mean ± SEM, *n* = 3 mice for each group.

It is well known that aging is associated with chronic inflammation (Lopez-Otin et al., 2013; Minciullo et al., 2016). Therefore, the gene expression of inflammation and anti-inflammation cytokines was detected by qPCR. Compared with young control, the mRNA levels of inflammatory factor including IL-1β, TNF-α, and IL-6 were significantly upregulated in the aging control mice, while acer truncatum seed oil markedly reversed the decreased mRNA levels of these pro-inflammatory factors in aging mice (Figures 6A–C). However, the oil had no effect on gene expression of IL-1α, which remarkably increased in the aging control (Figure 6D). Meanwhile, the oil treatment had no effects in the mRNA levels of anti-inflammatory cytokine IL-4 and IL-13 in aging mice (Figures 6E,F). These results suggest that acer truncatum seed oil alleviated inflammatory response in aging mice.

## Discussion

In the present study, we found that acer truncatum seed oil improved learning and memory of aging mice, with multiple mechanisms including activation of BDNF/TrkB signaling pathway, up-regulated postsynaptic related proteins expression, and down-regulated inflammatory factors mRNA level. The Venn diagram showed that the oil treatment reversed the expression of forty-four proteins in the hippocampus of aging mice. MCODE analysis indicated the DE proteins in the oil treated aging mice were related to complement and coagulation cascades, inflammatory response and innate immune response. Moreover, we found that 109 proteins were significantly up-regulated between the aging control mice and young control mice, while only 12 proteins were down-regulated. These aging-related proteins were primarily involved in synapse, oxidative stress, inflammation, mitochondria, and apoptosis process, etc.

Aging is a strong risk factor for multiple diseases including stroke, some aging-associated cancers, neurodegeneration, osteoarthritis, macular degeneration, and many other diseases. It remained a challenge to find effective drugs for the prevention and amelioration of aging. Rapamycin, senolytics, and metformin were currently under investigation in human trials for their potential to increase healthspan and lifespan (Campisi et al., 2019). Rapamycin extended the lifespan of yeast and increased maximum lifespan in mice by inhibiting the target of rapamycin (TOR) pathway (Harrison et al., 2009; Anisimov et al., 2011; Bitto et al., 2016). Metformin targeted several molecular mechanisms of aging, and also reduced the incidence of cancer and neurodegenerative disease (Barzilai et al., 2016). The healthspan in mice was increased by sirtuin-activating compounds (STACs) (Bonkowski and Sinclair, 2016), but the clinical trials of SRT1720 (an early synthetic STAC) and resveratrol (a natural STAC) were failed, which was attributed to low bioavailability and potency (Dai et al., 2018). Although NAD^+^ precursors existed protective activity against multiple aging-associated diseases in the animal models, the efficacy effects for humans have not been shown thus far (Martens et al., 2018).

Diet therapy is considered to be a promising approach to reverse or delay aging. Polyunsaturated acids such as DHA and omega-3, have been widely reported to improve learning and memory on animals, but marine organisms are the major source for DHA and Omega-3 (Cholewski et al., 2018). The oil used in this study is derived from the plant seed of acer truncatum, which is easier to obtain in high quantity. In addition to DHA and Omega-3, acer truncatum seed oil is also rich in linoleic acid, erucic acid, oleic acid, nervonic acids and VE, etc. Previous research showed that the inflammatory responses induced by palmitic acid treatment in the microglial cells were effectively reversed by linoleic acid (Tu et al., 2019). DHA diet in the Sprague-Dawley rats increased the expression level of BDNF, which acted on TrkB receptor signaling to attenuate learning disability in rats (Wu et al., 2008). Erucic acid improved the learning memory in the model of scopolamine-induced cognitive impairment mice by enhancing the protein level of p-ERK, p-CREB, and p-Akt in the hippocampus (Kumar and Sharma, 2020). Age-related cognitive impairment and onset of AD were prevented by given oleic acid supplements in humans (Snowden et al., 2017). Malania oleifera Chun oil rich in nervonic acid treatment markedly improved learning and memory of mouse models, which could better improve by the addition of DHA and VE (Wu R. et al., 2020). Moreover, antioxidant VE played an essential role in protecting cellular membrane-associated PUFAs from oxidative damage (Atkinson et al., 2010). Therefore, acer truncatum seed oil that has multiple effects, may be a better choice for anti-aging than DHA or Omega-3. Furthermore, we found that the seed oil had no toxic effects on tissues (Supplementary Figure 4). As expected, BDNF and TrkB was activated by acer truncatum seed oil treatment in aging mice. Moreover, postsynaptic proteins including PSD95, GluA1, and NMDAR1 were increased. The p-CREB was sensitive to the concentration of the BDNF, which regulated the transcription of specific target genes by BDNF (Asanuma et al., 1996; Chen et al., 2001). The level of p-CREB/CREB was significantly up-regulated by acer truncatum seed oil in the aging mouse. In general, CREB/BDNF pathway may underlie the acer truncatum seed oil elevated expression of synaptic proteins in the aging mice to alleviate cognitive impairment.

Aging and age-related diseases share basic mechanisms largely involving in the inflammation (Ferrucci and Fabbri, 2018). Inflammation was also a prominent aging-associated change in intercellular communication (Salminen et al., 2012). Indeed, inflammation had recently been proposed as one of the important symptoms of aging (Zhang et al., 2017). By MCODE analysis, we found that these DE proteins by seed oil treated aging mice vs. aging control mice were involved in innate immune response, inflammatory response pathway and complement and coagulation cascades. Moreover, the gene expression levels of pro-inflammatory factors including IL-1β, IL-6, and TNF-α were rescued by acer truncatum seed oil administration. Thus, the oil may alleviate aging by decreasing the inflammation.

Although only 61 DE proteins were identified in the oil-treated aging mice, 44 up-regulated DE proteins in the aging control were markedly recovered to approximate levels of the young control. These DE proteins were associated with the biological process including cellular response to interferon-beta, vitamin transport, and response to nutrient, etc. Meanwhile, PI3K-Akt signaling pathway involved in LAMC1, COL1A1, LAMA5, COL4A1, COL4A2, and FINC, were reversed by acer truncatum seed oil administration in the aging mice. Previous results explored that the PI3K-Akt pathway could regulate the replicative senescence of human vascular smooth muscle cells (Tan et al., 2016). Radix Astragali and Radix Astragali preparata exerted anti-aging function via network pharmacology and PI3K-Akt signaling pathway (Gong et al., 2021). The activity of PI3K/Akt signaling influencing neuronal survival and synaptic plasticity was declined in the aging rats of brain (van der Heide et al., 2006; Jiang et al., 2013). BDNF/TrkB signaling pathway performs a wide range of biological functions including inflammatory response and postsynaptic related proteins (Zhang et al., 2019). The increased expression of C1q in the prefrontal cortex of adult offspring after maternal immune activation was prevented by activation of TrkB (Han et al., 2017). Akt could also be activated by BDNF/TrkB (Longo and Massa, 2013). Therefore, BDNF/TrkB signaling pathway may be a key factor for the improvement effects of acer truncatum seed oil. In the next step, we will use BDNF receptor inhibitor K252a to inactivate TrkB, and then investigate whether acer truncatum seed oil treatment still has the improved effects on aging mice.

Proteomics of the hippocampus also showed that there were 121 DE proteins in the aging mice, which mainly involved in synapse, mitochondria, oxidative stress, and apoptosis, etc. Moreover, MCODE analysis revealed that the significant module identified from the PPI network including metabolism, cell migration, PI3K-Akt signaling pathway, mitophagy and complement activation, etc. These findings may become the basis for regulating protein expression levels to delay or reverse aging.

In conclusion, we discovered that acer truncatum seed oil improved cognitive deficits of aging mice by multiple pathways containing inflammation, synapse, BDNF/TrkB signaling, and PI3K-Akt signaling pathway. As a dietary supplement, acer truncatum seed oil, containing a variety of unsaturated fatty acids, β-Carotene and VE, would be a potential and effective choice for aging treatment.

## Data Availability Statement

The datasets presented in this study can be found in online repositories. The names of the repository/repositories and accession number(s) can be found below: The LC-MS/MS data of proteomics data have been deposited to the ProteomeXchange Consortium (http://proteomecentral.proteomexchange.org) via the iProX partner repository with the dataset identifier PXD024948.

## Ethics Statement

The animal study was reviewed and approved by the Ethics Committee of the Shenzhen Center for Disease Control and Prevention.

## Author Contributions

XY and GL conceived the project, designed the experiments, and wrote the manuscript. XL and TL designed and performed most of the experiments. TL performed the behavioral tests. XH analyzed the data and performed the statistics. JL performed the proteomics analysis. All authors contributed to the article and approved the submitted version.

## Conflict of Interest

The authors declare that the research was conducted in the absence of any commercial or financial relationships that could be construed as a potential conflict of interest.

## Footnotes

1 https://david.ncifcrf.gov/home.jsp

## Supplementary Material

Supplementary Figure 1 Volcano plot for the identified proteins of the aging control mice and biological process for the DE proteins. **(A)** Volcano plot for the identified proteins of the aging control mouse and **(B)** enrichment analysis by biological process.

Supplementary Figure 2 MCODE analysis for the DE proteins of the aging control mice vs. the young control mice. Detected PPI modules for aging related proteins.

Supplementary Figure 3 Volcano plot for the identified proteins of the oil-treated aging mice and biological process for forty-four proteins reversed by seed oil treatment in the aging mice. **(A)** Volcano plot for the identified proteins of the oil-treated aging mice and **(B)** enrichment analysis by biological process.

Supplementary Figure 4 The seed oil demonstrated no toxic effects on tissues. HE staining of the slice from heart, liver, spleen, kidney, and lung of aging mice with oil treatment or not.

Supplementary Table 1 Differentially expressed proteins between the aging control mice and young control mice.

Supplementary Table 2 Differentially expressed proteins between the aging mice treated with acer truncatum seed oil and aging control mice.
