# Supplementary figures and images for: Acer Truncatum Seed Oil Alleviates Learning and Memory Impairments of Aging Mice
Source: Front Cell Dev Biol. 2021 May 14;9:680386. doi: 10.3389/fcell.2021.680386 (PMC8160100; doi:10.3389/fcell.2021.680386)

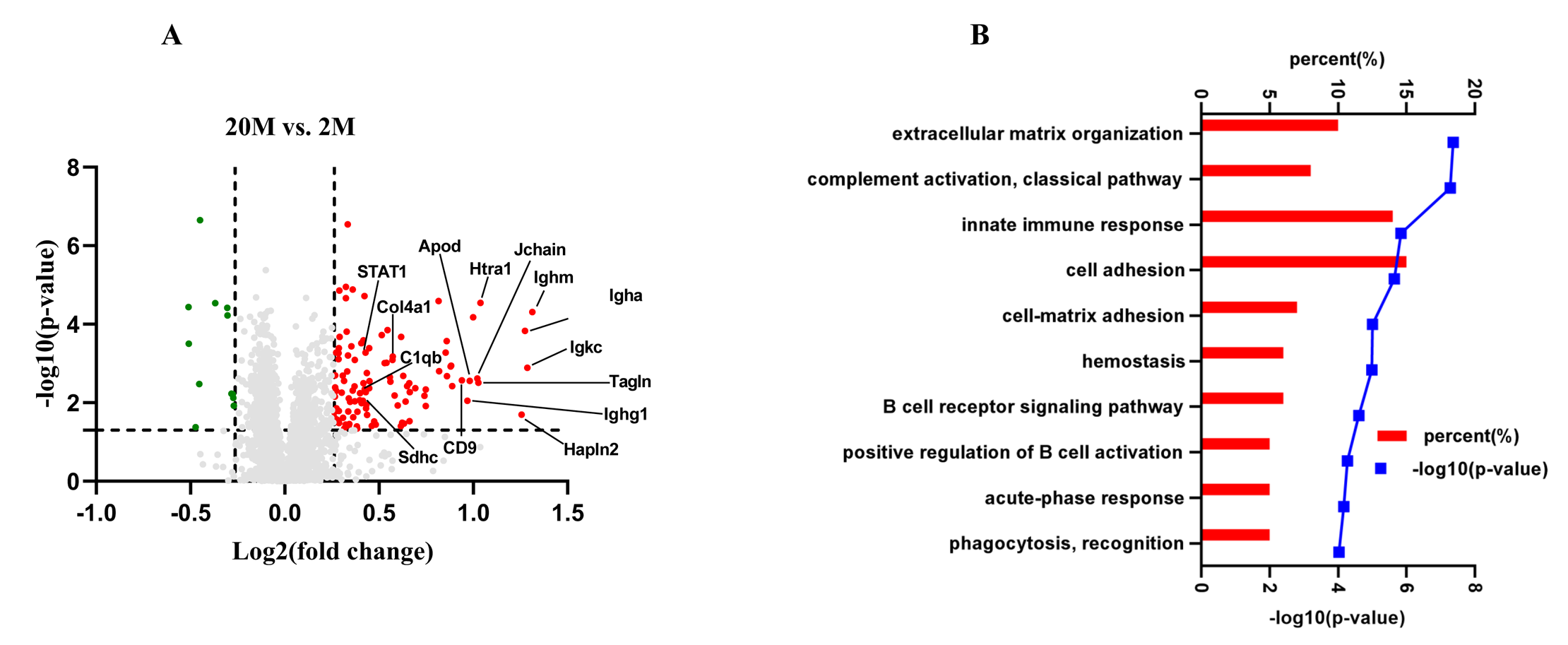

Supplement: Supplementary Figure 1 — Volcano plot for the identified proteins of the aging control mice and biological process for the DE proteins. (A) Volcano plot for the identified proteins of the aging control mouse and (B) enrichment analysis by biological process. [file Image_1.TIF]

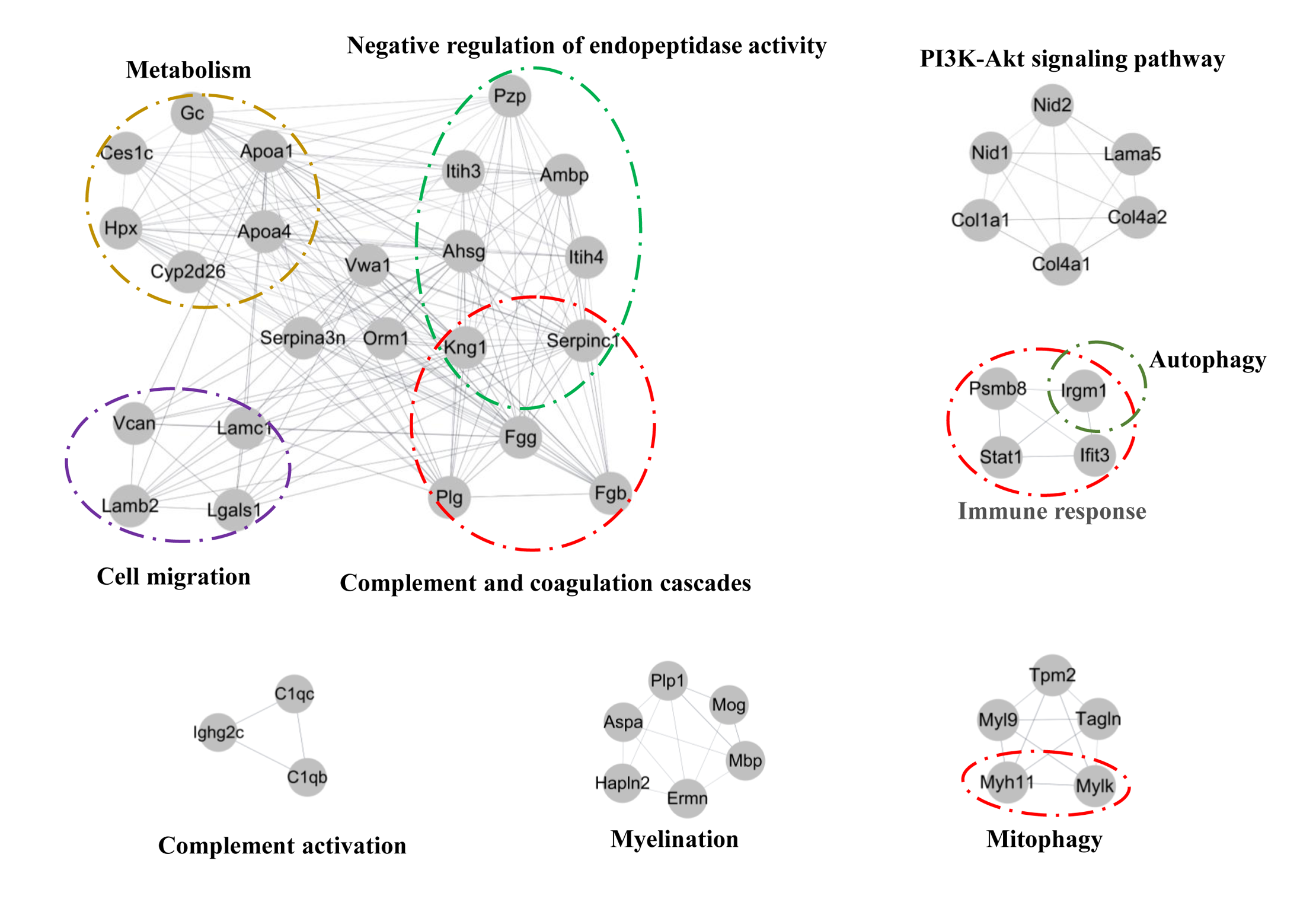

Supplement: Supplementary Figure 2 — MCODE analysis for the DE proteins of the aging control mice vs. the young control mice. Detected PPI modules for aging related proteins. [file Image_2.TIF]

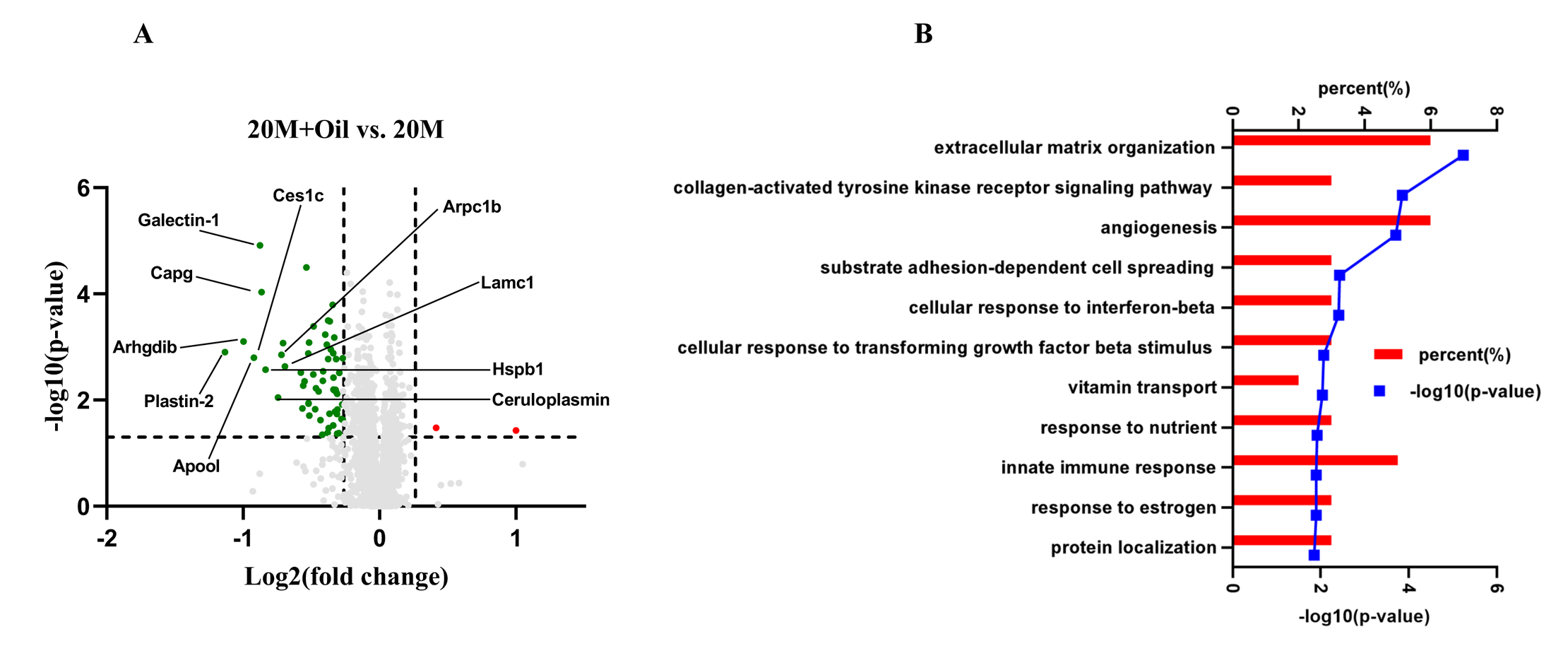

Supplement: Supplementary Figure 3 — Volcano plot for the identified proteins of the oil-treated aging mice and biological process for forty-four proteins reversed by seed oil treatment in the aging mice. (A) Volcano plot for the identified proteins of the oil-treated aging mice and (B) enrichment analysis by biological process. [file Image_3.TIF]

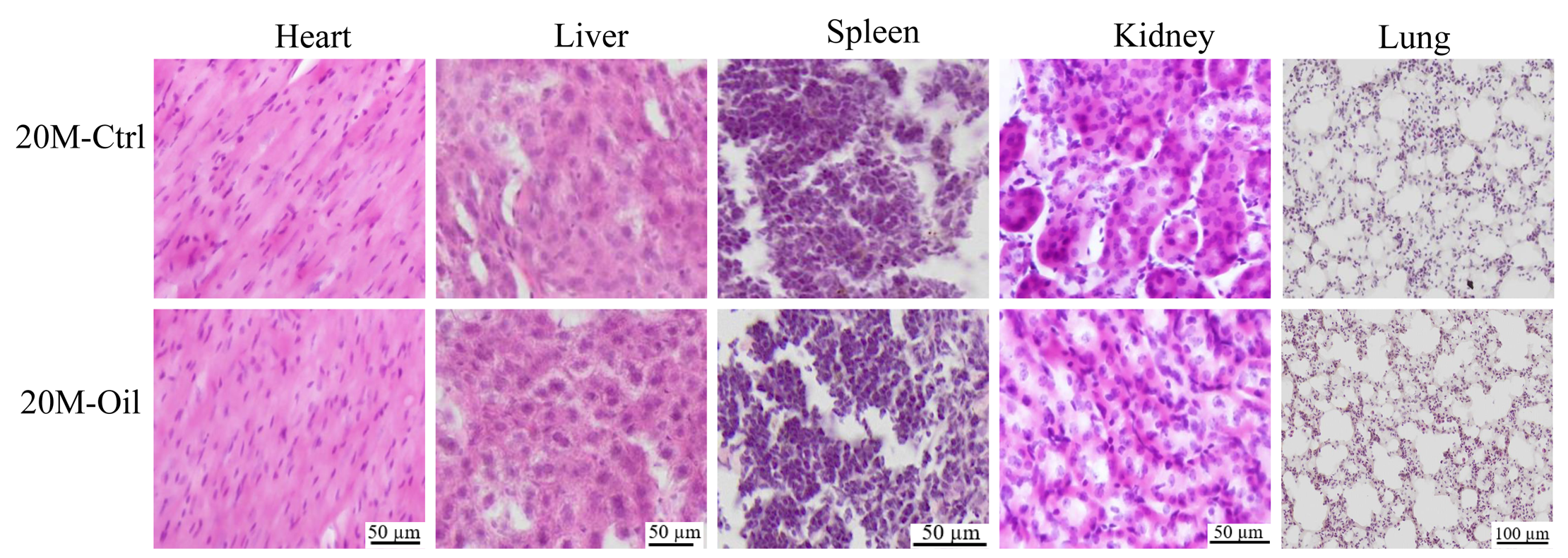

Supplement: Supplementary Figure 4 — The seed oil demonstrated no toxic effects on tissues. HE staining of the slice from heart, liver, spleen, kidney, and lung of aging mice with oil treatment or not. [file Image_4.TIF]
